# Content, Readability, and Accountability of Online Health Information for Patients Regarding Blue Light and Impact on Ocular Health

**DOI:** 10.7759/cureus.38715

**Published:** 2023-05-08

**Authors:** Parth Patel, Prem Patel, Harris Ahmed, Sila Bal, Grayson Armstrong, Jayanth Sridhar

**Affiliations:** 1 Ophthalmology, Augusta University Medical College of Georgia, Augusta, USA; 2 Ophthalmology, University of Texas Southwestern Medical School, Dallas, USA; 3 Ophthalmology, Loma Linda University Medical Center, Loma Linda, USA; 4 Ophthalmology, Massachusetts Eye and Ear Infirmary, Boston, USA; 5 Ophthalmology, Harvard University, Cambridge, USA; 6 Ophthalmology, Bascom Palmer Eye Institute, Miami, USA

**Keywords:** health communication, health education, readability, patient education, blue light

## Abstract

Objective

To evaluate the quality and readability of online health content regarding the ocular health effects of blue light.

Methods

Five commercial and five non-commercial websites with content regarding the ocular effect of blue light were examined. Quality evaluations were conducted using a 14-question assessment composed by the authors and the 16-question DISCERN instrument. Website accountability was evaluated via the Journal of the American Medical Association (JAMA) benchmarks. Readability was determined using an online tool (Readable). Correlational and comparative analyses were conducted where appropriate.

Results

The average questionnaire score was 84 (standard deviation [SD] ± 17.89, 95% confidence interval [CI] 77.32-90.68) out of 136 points (61.8%). Significant differences in quality were identified between websites (p = 0.02), with Healthline achieving the highest score. Compared to commercial websites, non-commercial websites trended toward having significantly higher median questionnaire scores (p = 0.06). Zero websites achieved all four JAMA benchmarks. The average reading grade level of content was 10.43 (SD ± 1.15, 95% CI 9.60 - 11.25), with differences between websites trending toward significance (p = 0.09). There was no correlation between resource readability and quality (ρ = 0.28; p = 0.43) or accountability (ρ = 0.47; p = 0.17).

Conclusions

There remain substantial deficiencies in the quality, accountability, and readability of online content concerning the effect of blue light on ocular health. Clinicians and patients must recognize such issues when recommending and consuming these resources.

## Introduction

Over the past few decades, there has been significant discussion regarding the effects of blue light on ocular health [1-3]. Media and major healthcare organizations have devoted extensive attention to potential blue light hazards in the eye, particularly on the retina [1-3]. In turn, manufacturers have advertised the purported benefits of blue light filtering lenses to generate a robust market for their products [4]. However, no research thus far has provided definitive evidence of the adverse effects of blue light exposure on the eye [5,6]. Some investigators have explored this question in depth, finding no impact of blue light exposure on the progression of chronic retinal diseases such as age-related macular degeneration (AMD) [5,6]. There are studies showing the benefit of blue light on various biological processes such as sleep cycle regulation, mood enhancement, and others [7-9].

For individuals striving to understand blue light-filtering lenses and their potential utility, online resources often serve as educational resources. Indeed, up to 70% of adults in the United States report using the Internet to gather research on medical conditions [10]. Although physicians remain an essential source of health-related information, numerous patients rate the information acquired from online searches as comparable or superior to that from a medical doctor [11]. Open discussions around online patient education materials (PEMs) can foster an improved patient-physician relationship [12,13]. Other reported benefits include patient empowerment, reduction of anxiety and depression, and increased participation in care [14-16].

There are challenges for patients seeking educational resources, particularly concerning ease of interpretation. Findings from the most recent National Assessment of Adult Literacy (NAAL) demonstrated that 43% of adult participants possessed health literacy skills at or below a basic level [17]. While the NAAL recommends that health information is written at the 6th-grade level or lower, a substantial corpus of literature indicates that this is, in fact, not the case for most PEMs [18]. Investigations of ophthalmologic topics ranging from cataracts to diabetic retinopathy to uveitis medications have demonstrated that the reading level of online PEMs consistently exceeds recommended guidelines [19-21]. As blue light continues to be a popular topic among online searches, this study aimed to evaluate the quality and readability of content focused on the ocular health effects of blue light from available resources on the Internet [22].

## Materials and methods

As a retrospective review of online data, this study was exempt from institutional review board review as a retrospective review of online data per our institutional standards. All research adhered to the tenets of the Declaration of Helsinki. Evaluated websites were selected based on resources accessible to patients who could complete a Google search using the “blue light” search term. A Google search was conducted on March 8th, 2022, to identify the top five results for academic/medical/non-profit and commercial resources. A total of 10 websites were included. Non-commercial websites were the American Academy of Ophthalmology (AAO) (https://www.aao.org/eye-health/tips-prevention/should-you-be-worried-about-blue-light), Harvard Health (https://www.health.harvard.edu/staying-healthy/blue-light-has-a-dark-side), Healthline (https://www.healthline.com/health/what-is-blue-light), Prevent Blindness (https://preventblindness.org/blue-light-and-your-eyes/), and WebMD (https://www.webmd.com/eye-health/blue-light-health), while commercial websites were All About Vision (https://www.allaboutvision.com/cvs/blue-light.htm), BluTech Lenses (https://blutechlenses.com/blog/what-is-blue-light/), EyeSafe (https://eyesafe.com/bluelight/), Thorne (https://www.thorne.com/take-5-daily/article/what-is-blue-light-and-how-does-it-affect-me), and ViewSonic (https://www.viewsonic.com/library/business/blue-light-filter-eye-strain/). PubMed (https://pubmed.ncbi.nlm.nih.gov) was utilized as the “gold standard” for peer-reviewed resources containing information about blue light.

After discussion among the authors, a 14-question assessment was designed to assess the resources (Table 1).

**Table 1:** Questionnaire for the content analysis of websites AAO: American Academy of Ophthalmology

	AAO	All About Vision	BluTech Lenses	EyeSafe	Harvard Health	Healthline	Prevent Blindness	Thorne	ViewSonic	WebMD
1. What is blue light?	2.67	4.00	2.67	3.67	2.00	3.67	4.00	4.00	4.00	3.33
2. How is blue light different from other types of light?	2.00	4.00	0.67	2.33	2.00	3.67	3.33	3.67	3.67	3.33
3. What are the sources of blue light?	3.33	3.67	3.33	3.67	3.33	3.67	3.67	4.00	4.00	4.00
4. Are there potential benefits to blue light?	3.00	3.33	3.00	3.33	2.67	4.00	4.00	3.00	3.67	2.33
5. Are there dangers to blue light?	3.33	3.33	2.67	3.33	3.33	3.67	3.00	3.33	3.33	3.33
6. What are ways people deal with blue light (e.g., glasses, software, etc.,)?	3.00	3.00	3.33	2.00	4.00	3.67	3.67	3.33	3.67	2.00
7. Should I block blue light exposure?	3.00	3.00	2.33	3.00	3.00	3.67	2.67	2.67	3.00	3.00
8. What are some ophthalmic symptoms associated with blue light?	2.33	3.00	2.67	2.67	1.67	3.33	3.33	2.67	3.33	3.00
9. Are ophthalmic diseases associated with blue light?	2.67	3.00	2.00	3.00	2.00	3.67	3.33	2.67	3.00	2.67
10. Can blue light cause cancer?	1.33	0.67	0.67	0.67	1.33	1.67	0.67	0.67	0.67	3.33
11. How does blue light affect sleep?	3.67	3.33	2.00	2.67	3.33	4.00	3.33	3.33	3.33	4.00
12. Is blue light bad for kids?	3.00	0.67	0.67	2.67	0.67	0.67	1.33	0.67	0.67	3.33
13. What does blue light do to the skin?	0.67	1.33	0.67	0.67	0.67	2.67	0.67	0.67	0.67	0.67
14. Is a physical filter better than software?	0.67	0.67	0.67	0.67	1.33	1.33	1.33	0.67	1.67	0.67
15. Are the aims clear?	3.67	4.00	3.33	3.67	3.67	4.00	3.67	3.33	4.33	3.67
16. Does it achieve its aims?	3.67	3.67	3.00	3.67	3.67	4.67	4.33	3.33	3.67	4.00
17. Is it relevant?	4.00	4.33	3.67	4.33	4.33	4.33	4.33	3.67	4.33	4.00
18. Is it clear what sources of information were used to compile the publication (other than the author or producer)?	3.00	2.67	1.00	3.33	3.00	5.00	3.67	2.00	1.00	3.00
19. Is it clear when the information used or reported in the publication was produced?	3.00	3.00	1.00	2.00	3.00	5.00	2.33	2.33	1.67	3.67
20. Is it balanced and unbiased?	4.33	4.00	2.00	2.33	4.33	4.67	4.67	1.67	2.00	4.33
21. Does it provide details of additional sources of support and information?	3.00	3.00	1.00	3.00	3.00	4.67	2.67	1.33	1.33	2.67
22. Does it refer to areas of uncertainty?	3.33	2.67	1.67	2.33	3.67	4.67	2.67	1.67	2.67	3.33
23. Does it describe how each treatment works?	2.00	3.33	2.00	2.33	3.33	4.00	4.33	3.33	3.33	1.67
24. Does it describe the benefits of each treatment?	2.33	3.67	2.00	2.33	3.00	4.33	3.33	3.00	3.67	1.67
25. Does it describe the risks of each treatment?	2.67	2.00	1.00	2.00	2.67	3.33	2.67	1.00	2.00	1.00
26. Does it describe what would happen if no treatment is used?	2.67	3.33	1.67	2.33	3.00	4.00	3.00	2.00	3.00	2.33
27. Does it describe how the treatment choices affect the overall quality of life?	1.33	2.33	1.33	2.00	3.00	3.00	2.00	2.00	3.00	1.67
28. Is it clear that there may be more than one possible treatment choice?	2.33	4.00	2.00	2.33	3.67	4.67	5.00	3.67	3.67	1.00
29. Does it provide support for shared decision-making?	1.00	4.00	1.67	3.67	3.00	3.33	4.00	1.67	3.67	1.00
30. Based on the answers to all of the above questions, rate the overall quality of the publication as a source of information about treatment choices	3.33	4.00	1.67	3.33	3.33	4.67	4.00	2.67	3.33	3.00
Total Points	80.33	91	57.33	79.33	85	111.67	95	74	85.33	81
Percentage (of 48)	59%	67%	42%	58%	63%	82%	70%	54%	63%	60%
Mean	2.68	3.03	1.91	2.64	2.83	3.72	3.17	2.47	2.84	2.7
SD	0.69	0.53	0.04	0.89	0.38	0.35	0.5	0.23	0.2	0.15
95% CI	0.98-4.38	1.72-4.35	1.82-2.01	0.44-4.85	1.90-3.77	2.86-4.58	1.91-4.42	1.89-3.05	2.35-3.34	2.34-3.06

The questions were derived from commonly encountered patient queries regarding blue light and were devised to evaluate the accuracy and breadth of available information. Websites were independently graded by three expert reviewers (G.W.A., S.B.B., J.S.). For each question, scores ranged from 0 to 4: 0 points suggested no relevant information was present; 1 point suggested information was inaccurate, unclear, lacked essential details, and/or was poorly organized; 2 points suggested partially complete information but with some lapses in details and organization; 3 points suggested essential information was present and organized appropriately; and 4 points suggested information was accurate, complete, and explained with purpose.

To corroborate our novel assessment, we additionally employed the DISCERN instrument. This 16-question tool has been well-validated in the literature to evaluate the quality of medical information [23]. For each question, expert reviewers rated resources on a scale of 1-5. A score of 1 indicated a definitive NO, whereas a score of 5 indicated a definitive YES; ratings between the two suggested the variable presence of question-specific elements.

Website accountability

The presence or absence of accountability was determined using the Journal of the American Medical Association (JAMA) benchmarks established in 1997. These four criteria are essential: authorship (affiliations, relevant credentials), attribution or sources (clearly listed references), currency or date of update, and disclosures [24].

Website readability

Readability was examined using multiple validated measures available via the readable tool (https://readable.com). The Flesch Reading Ease (FRE) score, calculated using word and sentence length, ranges from 0 to 100, with higher scores indicating greater readability. As a reference, a score between 70 and 80 indicates a text readable by a 7th grader. Other included measures were the Flesch-Kincaid Grade Level, Coleman-Liau Index, Gunning Fog Index, and Simple Measure of Gobbledygook Index, which produce scores that directly correlate with US grade reading levels.

Statistical analysis

The Kruskal-Wallis H test compared assessment scores between websites with a post hoc Dunn’s test performed for individual pairwise comparisons. Differences between academic and commercial resources were analyzed using the Mann-Whitney U test. Assessments of interobserver reliability and correlation were conducted through Spearman’s ρ. Statistical tests were completed using GraphPad Prism 9.0 (San Diego, CA), and significance was set at p < 0.05.

## Results

Analysis of website content

Our investigation analyzed ten websites, of which only Prevent Blindness offered the options to enable text reading, alter font size, and reverse contrast. No other websites offered similar accessibility features for readers. Five of 10 websites (50%) provide a relevant graphic, although their quality was not examined. Interobserver reliability was moderate to strong between all three reviewers (ρ = 0.98 between J.S. and S.B.B., p < 0.001; ρ = 0.63 between J.S. and G.W.A, p = 0.05; ρ = 0.57 between S.B.B. and G.W.A., p = 0.09).

Among all websites, the average score was 84.00 (SD ± 17.89, 95% CI 77.32 - 90.68) of 136 potential points (61.8%). With the inclusion of PubMed, significant differences in the completeness and quality of websites were observed (H = 21.92; p = 0.02). With the exclusion of PubMed, Healthline had the highest average questionnaire score, with 111.7 points (82.1%), whereas BluTech had the lowest, with 57.3 points (42.1%). The average scores for each resource are presented in Table 2.

**Table 2:** Content analysis of the websites AAO: American Academy of Ophthalmology

	Total Points	Percentage (of 136)	Mean	SD	95% CI
AAO	80.33	59%	2.68	0.69	0.98-4.38
All About Vision	91.00	67%	3.03	0.53	1.72-4.35
BluTech Lenses	57.33	42%	1.91	0.04	1.82-2.01
EyeSafe	79.33	58%	2.64	0.89	0.44-4.85
Harvard Health	85.00	63%	2.83	0.38	1.90-3.77
Healthline	111.67	82%	3.72	0.35	2.86-4.58
Prevent Blindness	95.00	70%	3.17	0.50	1.91-4.42
Thorne	74.00	54%	2.47	0.23	1.89-3.05
ViewSonic	85.33	63%	2.84	0.20	2.35-3.34
WebMD	81.00	60%	2.70	0.15	2.34-3.06

A significant difference in scores was noted between BluTech and both PubMed (H = 29.50; p = 0.01) and Healthline (H = 26.17; p < 0.05). Non-commercial websites tended to have significantly higher median questionnaire scores than their commercial counterparts (86.0 vs. 79.0; p = 0.06).

Analysis of website accountability

Of all resources, zero attained all four JAMA criteria, and three (30%) non-commercial attained three (Table 3). Alternatively, two websites (20%), which were commercial, achieved none of these benchmarks. The most fulfilled benchmark was currency (eight [80%]). Resource content quality and accountability were not significantly correlated (ρ = 0.47; p = 0.17).

**Table 3:** Accountability analysis of the websites

JAMA Criteria	n (%)
4 Benchmarks	0 (0%)
3 Benchmarks	3 (30.0%)
2 Benchmarks	2 (20.0%)
1 Benchmark	3 (30.0%)
0 Benchmarks	2 (20.0%)
Attribution	2 (20.0%)
Authorship	5 (50.0%)
Currency	8 (80.0%)
Disclosure	1 (10.0%)

Analysis of website readability

The average FRE score was 61.85 (SD ± 5.01, 95% CI 58.27-65.43), and the average reading grade level was 10.43 (SD ± 1.15, 95% CI 9.60-11.25). These measures exhibited a robust correlation (ρ = -0.96, p < 0.001).

The average reading grade level difference trended toward significance across resources (H = 15.05; p = 0.09), as indicated in Table 4.

**Table 4:** Readability analysis of the websites AAO: American Academy of Ophthalmology

	AAO	All About Vision	BluTech Lenses	EyeSafe	Harvard Health	Healthline	Prevent Blindness	Thorne	ViewSonic	WebMD
Flesch Reading Ease	66.2	53.4	64.4	61.2	58.4	61.3	65.9	62.3	55.7	69.7
Average Reading Grade Level	9.85	12.50	10.08	9.75	11.33	10.58	9.70	10.15	11.78	8.55
Average Reading Grade Level, SD	1.36	1.73	1.73	1.74	1.31	1.60	1.72	1.43	1.42	1.67
Average Reading Grade Level, 95% CI	7.68-12.02	9.74-15.26	7.33-12.82	6.99-12.51	9.24-13.41	8.02-13.13	6.97-12.43	7.88-12.42	9.51-14.04	5.90-11.20

All About Vision had the lowest FRE score (53.4) and highest average reading grade level (12.5), suggesting poor readability. Conversely, WebMD had the highest FRE score (69.7) and lowest average reading grade level (8.55), suggesting fair readability. Website quality and readability were unrelated (ρ = 0.28; p = 0.43). No average reading grade level difference was identified between non-commercial and commercial resources (10.15 vs. 9.85; p = 0.31).

## Discussion

As many patients turn to the Internet for health information, accessing validated and comprehensible online resources is essential. In ophthalmology, numerous studies have investigated online content quality, accuracy, and readability, covering cataracts, AMD, diabetic retinopathy, epiretinal membranes, and glaucoma [19-21,25,26]. However, to our knowledge, this study is the first to assess these parameters in content focusing on “blue light,” which has been the subject of increasing public attention as evidenced by recent search trends [22]. Our study demonstrated significant variations and limitations in content, readability, and accountability of major medical websites covering blue light. Although no significant differences were observed between non-commercial and commercial sources, patients should consult their physician when seeking health information online, especially as our knowledge of blue light constantly evolves.

Our analysis suggested that no PEMs regarding blue light were written below the NAAL-suggested 6th-grade reading level for patients striving to become more involved with their healthcare [18]. This finding is consistent with the myriad of other investigations evaluating the readability of websites for other ophthalmic topics [19-21,25,26]. The Internet has become a primary repository of patient healthcare information [10]. Therefore, such analyses are essential for guiding clinicians when recommending appropriate patient resources.

Blue light is a popular topic of discussion for patients online due to the substantial marketing efforts from manufacturers of blue light-filtering lenses [3,4,22]. However, the claim that such products positively impact ocular health remains disputed in the ophthalmic literature [2,3,27]. Negative health impacts associated with blue light predominantly arise from experimental laboratory investigations that suggest short-wavelength light may stimulate retinal phototoxicity [6,28,29]. Nonetheless, a recent Cochrane review revealed uncertain evidence regarding any differences in the preservation of macular function or progression of AMD between blue light-filtering and non-blue light-filtering intraocular lenses (IOLs) among humans [30]. The evidence regarding blue light-filtering glasses is more tenuous [31]. There is a lack of high-quality clinical trials evaluating the efficacy of this intervention, with most literature suggesting an inconclusive benefit [31]. As prolonged use of computers and digital devices has become commonplace in modern society, it is unsurprising that many users complain of ocular symptoms, including eyestrain, diplopia, and blurred vision. The etiology of these symptoms, collectively denoted computer vision syndrome, is multifactorial and likely stems from reduced blinking and the subsequent disruption of the tear film, uncorrected refractive error, and oculomotor dysfunction, among other causes [32]. Therefore, it is difficult to ascertain the exact role of blue light in its pathogenesis.

In this uncertainty, online resources must provide a comprehensive, unbiased discussion of blue light and the utility of blue light-filtering lenses. Inaccurate or incomplete information can weaken the patient-provider relationship, leading to noncompliance with future clinical recommendations [33]. Unfortunately, the findings presented herein suggest substantial differences in the quality of the information provided by websites, with scores ranging from 57.33 to 111.7 (out of 136 maximum points). Healthline provided the highest quality information (111.7 out of 136 points), although its average readability (10.58) was remarkably higher than the recommended 6th-grade reading level, highlighting a significant barrier to comprehensibility. Furthermore, certain topics (e.g., the efficacy of a physical blue light filter versus a software blue light filter and the effects of blue light on the skin) were inadequately addressed across resources. These deficits emphasize the limitations of online resources for patients striving to understand blue light. In particular, it is necessary to note that commercial websites discussing blue light should be cautiously approached. Commercial resources tended to provide poorer quality information relative to their non-commercial counterparts, with BluTech Lenses, a commercial website, observed to have a notably lower questionnaire score (57.33).

The JAMA benchmark criteria were used to identify discrepancies in accountability. No websites attained all four criteria, and the majority (7 of 10) achieved two or fewer, indicating that resources discussing blue light possess poor accountability.

Further highlighting concerns with commercial websites was that 40% (2 of 5) did not fulfill any JAMA benchmarks. Whether this finding generalizes to other online commercial resources regarding blue light is only possible with a larger sample size.

Nevertheless, patients are encouraged to utilize non-commercial websites to avoid the potential pitfalls associated with their commercial counterparts. However, it is also important to note that even primary non-commercial sources, such as the AAO, did not fare well in our analysis. Of particular interest, there was no correlation between website accountability and the quality of provided information, a result mirrored among other investigations examining the content of online ophthalmic resources [19,20,25]. Accordingly, the lack of accountability observed here appears to be a ubiquitous problem for numerous websites dispensing medical information.

There are several limitations to our investigation. One component of the questionnaire utilized to evaluate the quality of resource content was derived from a discussion among the authors to identify common patient queries regarding blue light. Therefore, this assessment was unstandardized, unlike the DISCERN instrument that comprised the remaining questions. The sample of this study consisted of 10 websites, excluding other resources patients may potentially encounter and limiting generalizability. Including more websites could have increased the breadth of the study, although the sample size is consistent with similar evaluations of online ophthalmic content [19,20,25]. Interobserver reliability was mixed, although a strong trend indicated the need for greater readability and quality for the websites assessed. Furthermore, as the investigation was focused on blue light, the findings presented here cannot be reasonably extrapolated to other ophthalmic topics. Additionally, the content evaluated was mainly based in the United States, and our findings may not be as relevant to other countries. Lastly, it should be noted that patients also rely on clinic-based brochures as a source of information, and this arena was not captured or assessed by our analysis.

## Conclusions

There is significant variation in the quality and readability of freely available online content focused on the health effects of blue light. According to JAMA standards, many websites lack transparency regarding website accountability. Most websites are written at a reading grade level higher than that recommended by established guidelines. Since commercial stakeholders are incentivized to advertise eyeglasses that filter blue light from digital devices, patients should be encouraged to discuss the information they gather from various resources with their physicians. Additionally, physicians should strive for their own communication to be at the appropriate reading level when discussing blue light filters with patients.
